# Correlation between morphological features of the anterior cruciate ligament: A quantitative study using a porcine model

**DOI:** 10.3389/fvets.2023.1115068

**Published:** 2023-02-09

**Authors:** Huizhi Wang, Zhuoyue Zhang, Qinyi Shi, Yi-Ming Zeng, Cheng-Kung Cheng

**Affiliations:** ^1^School of Biomedical Engineering, Shanghai Jiao Tong University, Shanghai, China; ^2^School of Biological Science and Medical Engineering, Beihang University, Beijing, China; ^3^Shanghai Ninth People's Hospital, School of Medicine, Shanghai Jiao Tong University, Shanghai, China

**Keywords:** anterior cruciate ligament, ACL reconstruction, morphology, cross-sectional area, insertion site, length, quantitative correlation

## Abstract

**Introduction:**

Knowledge of the morphological features of the anterior cruciate ligament (ACL) is critical for accurate reconstruction of it. This study aimed to explore the quantitative correlations among different morphological features of the ACL, thus to provide useful information for improving anatomical reconstruction techniques and designing artificial ligaments.

**Methods:**

19 porcine knees were fixed at full extension using 10% formalin and were dissected to expose the ACL. ACL lengths were measured using a caliper. Mid-substances of the ACL were cut and scanned using X-ray microscopy, and the cross-sectional area (CSA) was measured at the isthmus. Margins of direct and indirect bone insertion sites were distinguished and marked. Measurements were performed on digital photographs to obtain the areas of bone insertions. Statistical analysis using nonlinear regression was used to identify potential correlations among the measurements.

**Results:**

The results showed that the CSA at the isthmus was significantly correlated with the total area of the bone insertion sites and the area of tibial insertion. The area of the tibial insertion was significantly correlated with the area of its direct insertion site. In contrast, the area of the femoral insertion was significantly correlated with the area of its indirect insertion site. The area of the indirect tibial insertion showed a weak correlation with the length of ACL, whereas the length of the ACL was not able to predict or be predicted by any other parameters.

**Conclusions:**

The CSA at the ACL isthmus is more representative for assessing the size of the ACL. However, ACL length has little correlation with the CSA of the isthmus or bone insertion sites, and thus should be evaluated independently for ACL reconstruction.

## 1. Introduction

**Figure 1:**
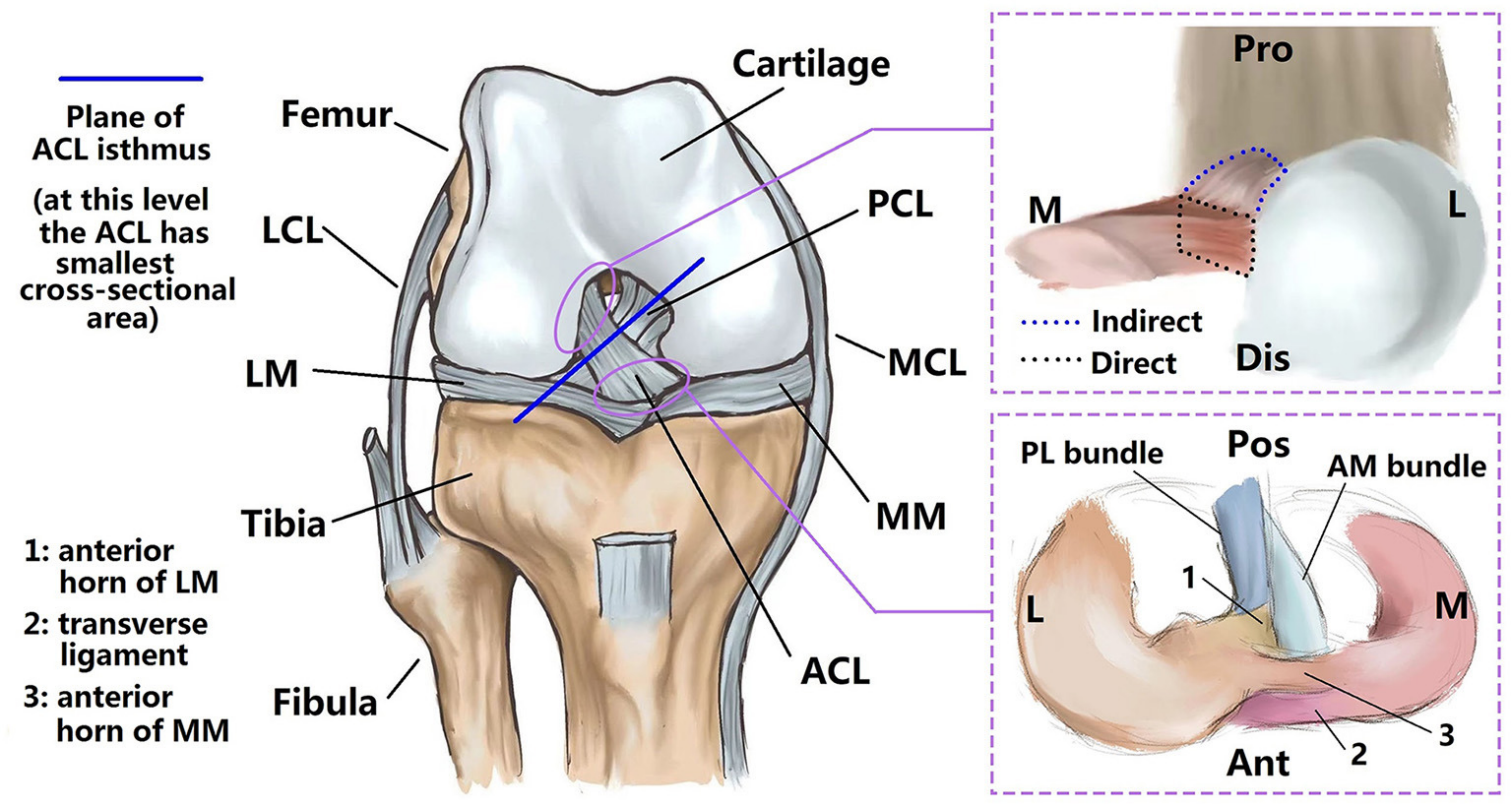
Sketch of intact knee joint. The ACL has a characteristic hourglass shape (fans out to its bony insertion sites) **(left)**. Bone insertions can be divided into direct and indirect types. The direct insertion connects to the midsubstance fibers, while the indirect insertion extends from the direct insertion to the bone, but is not directly connected with the midsubstance fibers **(upper right)**. The anterior horn of the lateral meniscus inserts into the ACL substance and passes through the interface between tibial insertion sites of the anteromedial and posterolateral bundles of the ACL **(lower right)**. LCL, lateral collateral ligament; LM, lateral meniscus; MM, medial meniscus; MCL, medial collateral ligament; PCL, posterior cruciate ligament; Pro, proximal; M, medial; L, lateral; Dis, distal; Pos, posterior; Ant, anterior.

The anterior cruciate ligament (ACL) is one of the most important ligaments in the knee joint and primarily acts to constrain excessive joint motions, particularly anterior tibial translation and internal and valgus tibial rotations (1). However, the ACL is commonly injured and has very little capability to regenerate, often requiring surgical reconstruction with grafts either to supplement or replace the native ligament. The native ACL has an hourglass shape with fan-like extensions to its bone insertion sites (Figure 1), which provides strength while avoiding impingement with the top of the intercondylar notch (2). In contrast, grafts have a more cylindrical shape and cannot be naturally anchored to the bone insertion sites in the same way as the native ligament. The width of the ACL isthmus (where the ACL has a smallest cross-sectional area, see Figure 1) in the coronal plane is typically used for choosing a graft size (3), while other morphological features such as cross-sectional area (CSA) of the isthmus, area of the insertion sites and ligament length are not taken into consideration. However, previous studies (4) have shown that insufficient reconstruction of the bony insertion sites can result in inadequate joint rotational stability and uneven articular contact stresses, which in the long term may lead to osteoarthritis. Therefore, closely replicating the anatomical features of the native ACL is crucial for proper knee functionality.

Quantitative studies on ACL morphology (3, 5–9) have shown wide variations between individuals (35.1–103.6 mm^2^ for cross-sectional area of ACL isthmus, 50.8–156.0 mm^2^ for the area of femoral insertion site, 31.4–175.8 mm^2^ for the area of tibial insertion site and 23.8–31.1 mm for ACL length), indicating that graft size should be correspondingly individualized for better restoration of ACL functionality. Fujimaki et al. (8) found that the ACL isthmus is located about half-way between the bone insertion sites, and the average CSA of the isthmus (39.9 ± 13.7 mm^2^) was found to be significantly smaller than the area of the femoral insertion site (122.1 ± 30.2 mm^2^) and tibial insertion site (175.8 ± 64.3 mm^2^). Similarly, Harner et al. (5) reported that the areas of femoral and tibial insertion sites could be over 3.5 times larger than the CSA of the ligament midsubstance. Fujimaki et al. (8) found that the ACL length reached a maximum at full extension and decreased with the flexion angle (31.1 ± 3.1 vs. 24.3 ± 3.2 mm at 0° and 90° of flexion). However, Harner et al. (10) stated that the joint flexion angle did not affect the CSA of the ACL but was noted to alter the cross-sectional shape. Furthermore, the concept of “direct” and “indirect” insertion sites of the ACL has been introduced (11–13), with the “indirect” part referring to a broader fan-like extension of the “direct” part (Figure 1). The indirect site is also not directly attached to the midsubstance fibers of the ACL. The area of the indirect insertion region on the femur is generally larger than the direct region (50.8 vs. 91.4 mm^2^) (7).

However, few studies have explored potential correlations among morphological features of the ACL. This study aimed to explore potential correlations between the CSA at the ACL isthmus, areas of bone insertion sites (direct, indirect, and total), and length of the ACL. It was hypothesized that the areas of the ACL bone insertion sites could be approximated by the area of the ACL isthmus and ACL length. The results of this study may provide a scientific basis for developing improved anatomical reconstruction techniques and for designing artificial ligaments. Any proven correlations may also provide surgeons with a reference dimension on the ligament when choosing a suitable graft size for ACL reconstruction. Porcine knees were used in this study because of their similarity to human knees in terms of bone density, morphology and biomechanical characteristics (14, 15).

## 2. Materials and methods

### 2.1. Sample preparation

**Figure 2:**
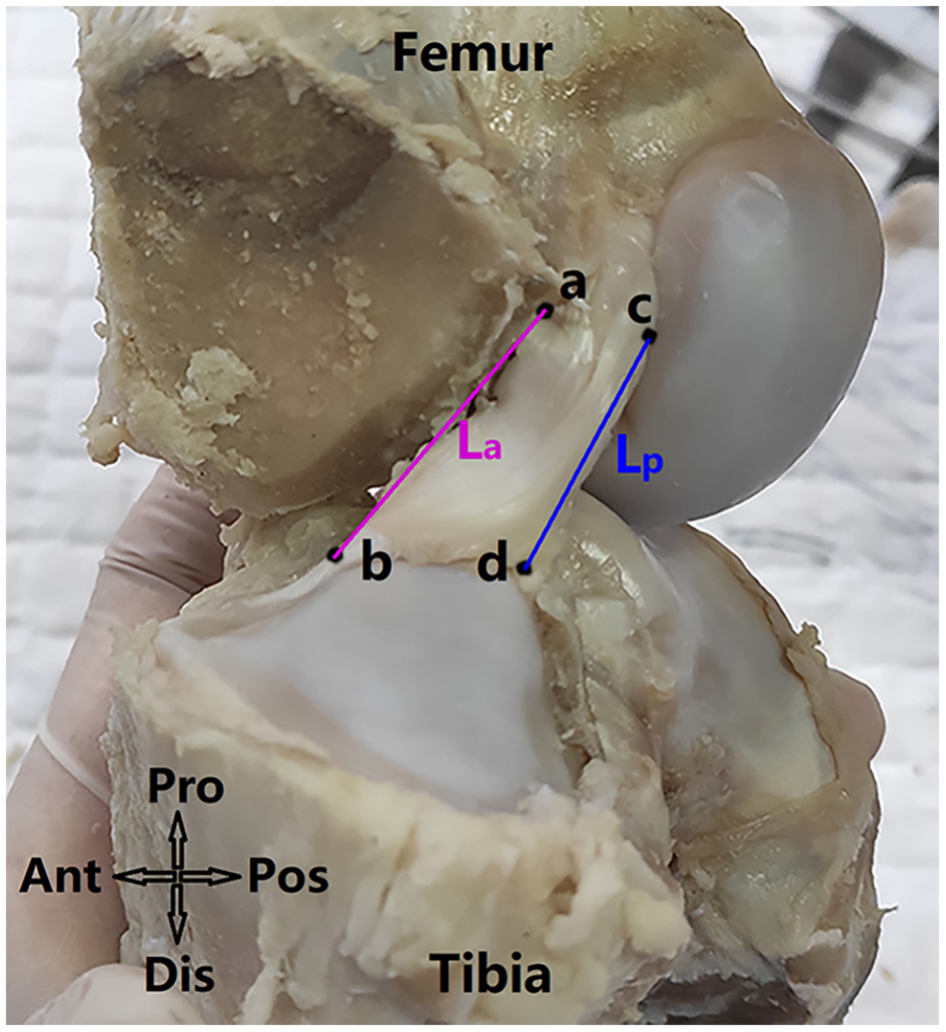
Measuring anterior and posterior lengths of the ACL (referred as *L*_*a*_ and *L*_*p*_ respectively). Anatomical directions referred to as Ant, anterior; Pos, posterior; Pro, proximal; Dis, distal. Point a and b refer to the anterior edge points of the ACL on the femoral and tibial direct insertion sites respectively and point c and d refer to the posterior edge points of the ACL on the femoral and tibial direct insertion sites. The ACL length (L) was defined as $L=\;\frac{L_{a}+L_{p}}{2}$.

The research protocol was approved by the university's Institutional Animal Care and Use Committee. 19 porcine knees from Duroc-landrace-yorkshire crossbred pigs (1 years old, all male) were purchased from a licensed abattoir and frozen within 12 h after death. Samples were thawed for 12 h at room temperature before dissecting. Tissues were removed 12 cm proximal and distal to the joint line and muscles and tendons were further removed to expose the joint capsule. Then the joint was fixed at full extension in 10% formalin at room temperature for 48 h (12). All ligaments (except for the ACL) and menisci and the medial femoral condyle were removed to expose the whole ACL (Figure 2). The knee was fixed at full extension because the ACL was straight at this position and the flexion of the knee resulted in an irregular shape of the ACL.

### 2.2. Measurement of ACL length

The anterior and posterior length of the ACL (*L*_*a*_ and *L*_*p*_ in Figure 2) were measured using an electric caliper (0.01 mm in precision). The ACL length (*L*) was calculated as $L=\;\frac{L_{a}+L_{p}}{2}$. Specifically, *L*_*a*_ was the line connecting the anterior edge points of the ACL at the femoral and tibial direct insertion sites (point *a* and point *b* respectively), and *L*_*p*_ was the line connecting the posterior edge points of the ACL at the femoral and tibial direct insertion sites (point *c* and point *d* respectively).

### 2.3. Measurement of CSA at ACL isthmus

**Figure 3:**
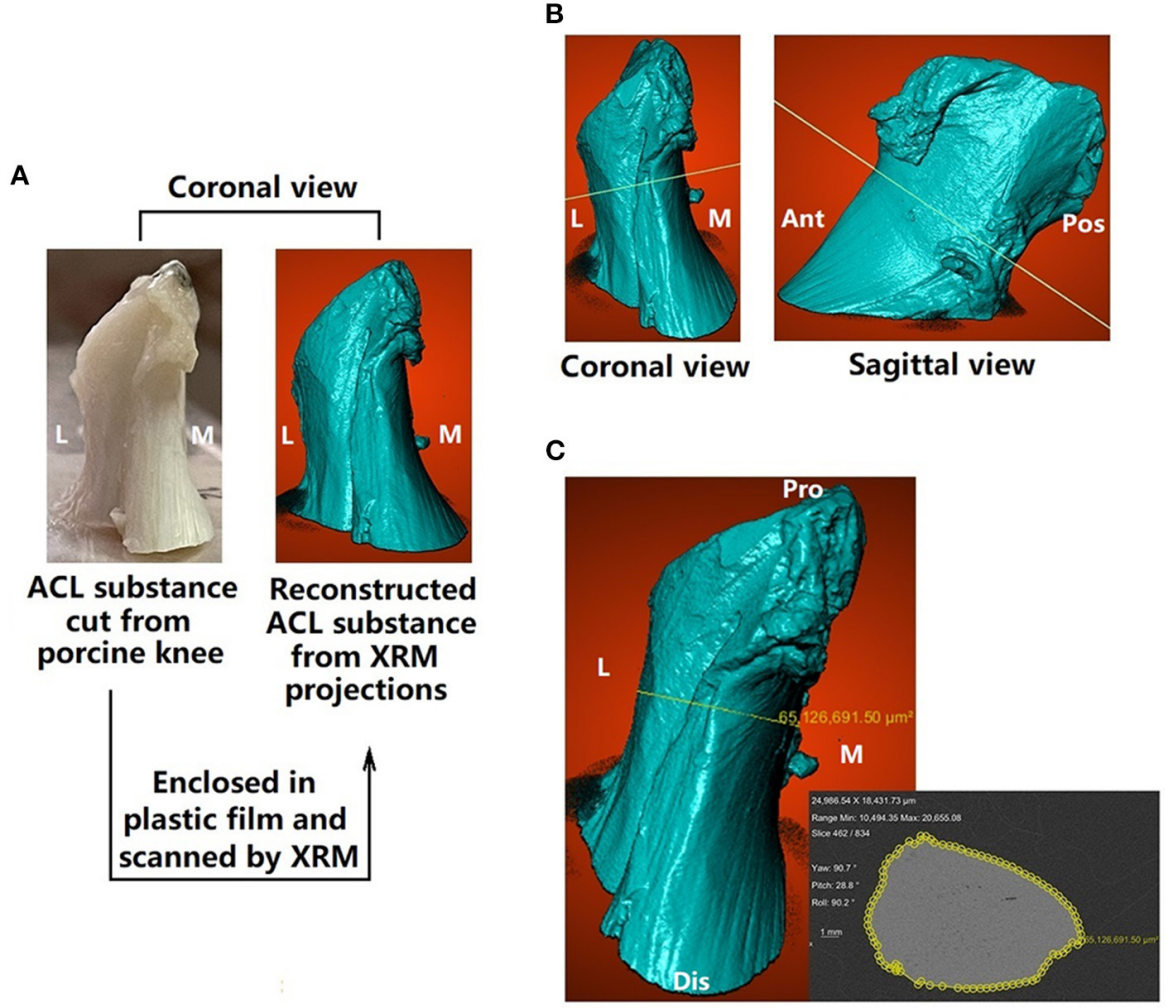
Measuring cross-sectional area (CSA) of the ACL. **(A)** ACL substance was cut from the porcine knee, enclosed in plastic film and scanned by x-ray microscopy (XRM) **(B)** a plane (isthmus plane) was found to simultaneously go through the ACL isthmus in the coronal and sagittal views **(C)** CSA at ACL isthmus (enclosed by yellow rings) was measured on the isthmus plane. M, medial; L, lateral; Ant, anterior; Pos, posterior; Pro, proximal; Dis, distal.

The ACL was cut along the surfaces of its femoral and tibial insertion sites. Next, the ACL substance was enveloped with plastic film and scanned by x-ray microscopy (Zeiss Xradia 520 Versa, Carl Zeiss AG, Germany) at a resolution of 30 × 30 × 30 μm. 1,101 projections were measured with an exposure of 1 s at 70 kV and 6 W. The sample-source distance was 81.8 mm and the sample-detector distance was 104.0 mm. The acquired projections were reconstructed with XMReconstructor (Figure 3A) and the CSA at the ACL isthmus was measured using Dragonfly software, provided with the scanner. As shown in Figure 3, a cross-sectional plane was found to simultaneously pass through the isthmus of the ACL on the coronal and sagittal plane (Figure 3B), from which the CSA could be measured using measuring tools provided by the Dragonfly software (Figure 3C).

### 2.4. Measurement of area of ACL insertion sites

**Figure 4:**
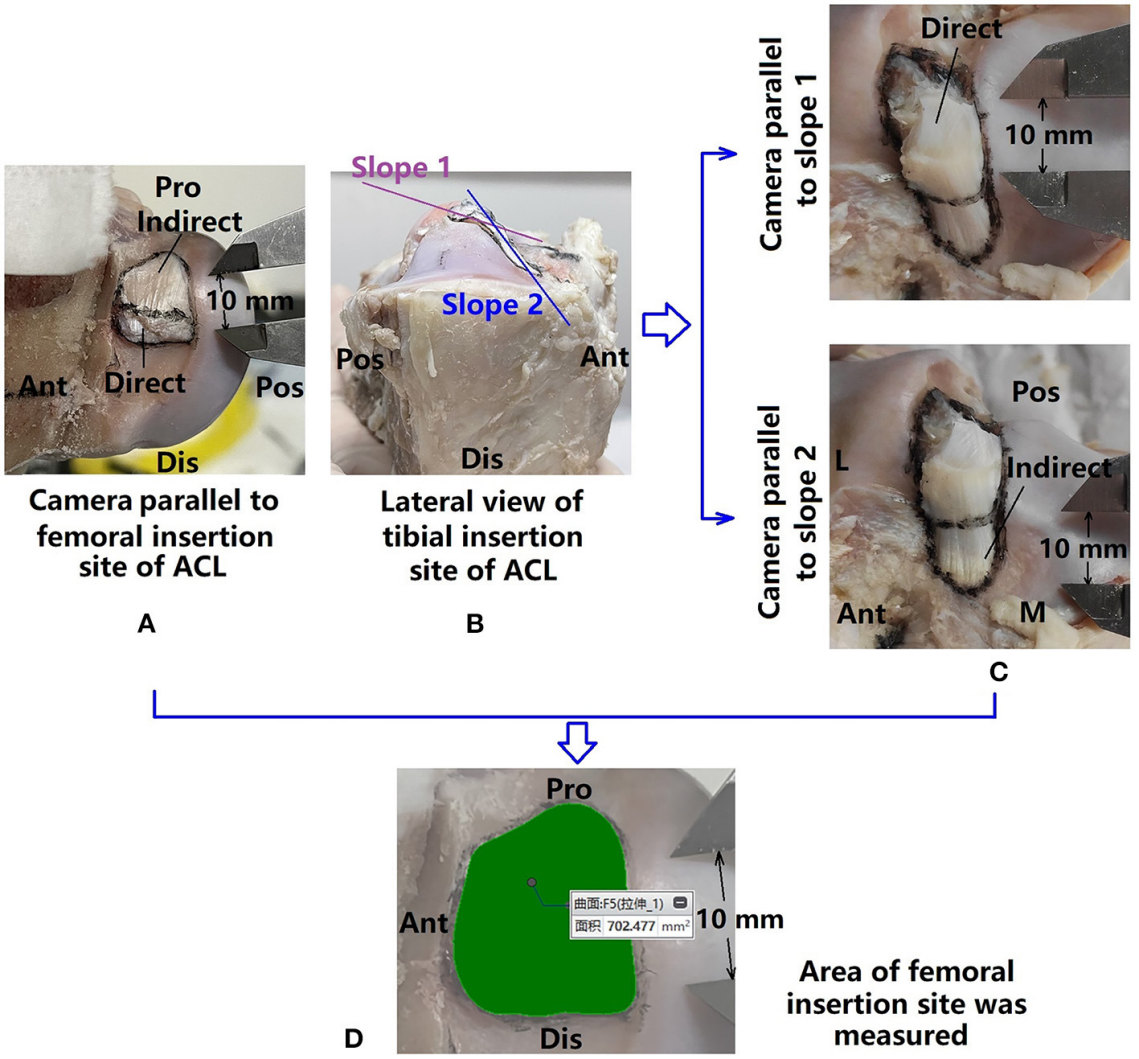
Measuring areas of ACL bone insertion sites. **(A)** Femoral insertion site marked and photographed; **(B)** tibial insertion site showing double-sloped shape, and thus **(C)** the tibial insertion site was photographed twice with the camera parallel to each slope; **(D)** marked areas were measured with a precision of 0.001 mm.

The circumference of the femoral and tibial insertion sites were marked with permanent marker (Figure 4C), as well as boundaries between the direct and indirect insertion sites. Digital photographs of the insertion sites were taken with an electric caliper placed near the circumferences. The camera was slightly tilted in all directions to find the largest projected area to represent the best approximation of the surface area of the insertion site. The femoral insertion site was found to be relatively planar but the tibial insertion site was double-sloped (Figures 4A, B). Thus, one photograph was taken for measuring the projection of the femoral insertion site while two were taken for measuring the tibial side.

Photographs were uploaded to a computer and the areas of the ACL insertion sites (direct, indirect, and whole) were measured in Creo Parametric 7.0 (PTC, Massachusetts, USA) (0.001 mm in precision) (Figure 4D).

### 2.5. Statistical analysis

All statistical analyses were performed using IBM SPSS Statistics 23 (SPSS Inc, Chicago, USA). Using power analysis, the required sample size was 19, with significance level of α = 0.05 and power level of β = 80%. Two independent observers carried out all measurements, and the first observer measured twice. The intra- and inter- observer reliabilities were calculated using the intraclass correlation coefficient (ICC) with a 2-way random model of absolute agreement. The ICC can assume any value from 0 to 1, where a value >0.80 represents good agreement, between 0.60 and 0.79 signifies moderate agreement, and 0.59 signifies poor agreement. Results are reported as average ± SD. Non-linear regression analysis was carried out between each two of the measured parameters. *P*-values of <0.05 were considered significant, and the equation representing the best-fit line and the *R*^2^ value representing consistency between the best-fit line and the raw data (also called the coefficient of determination) were calculated. Potential non-linear correlations between compound parameters (total area of femoral and tibial insertion sites (direct and total), ratio between the area of bone insertion to CSA, etc.) were also explored, including the correlations between: (i) CSA at ACL isthmus and the total area of all insertion sites; (ii) CSA at ACL isthmus and the total area of direct insertion sites; (iii) ratio between total area of the femoral insertion site to CSA at ACL isthmus and ratio between total area of tibial insertion site to CSA at ACL isthmus.

### 2.6. Ethics approval statement

All procedures performed in this study involving porcine knees were approved by the Institutional Animal Care and Use Committee in Shanghai Jiao Tong University (IACUC APPROVAL No. 202101260).

## 3. Results

The intraclass correlation coefficient (ICC) among intra- and inter- observer measurements is shown in Table 1. ICC values for all measurements were >0.80, revealing good measurement reliability between intra- and inter- observer groups.

**Table 1:** Intraclass correlation coefficient (ICC) among intra- and inter- observer measurements.

	**ICC (intra- observer)**	**ICC (Inter- observer)**
CSA at ACL isthmus	0.998^**^	0.995^**^
Area of direct femoral insertion	0.996^**^	0.997^**^
Area of indirect femoral insertion	0.998^**^	0.999^**^
Area of total femoral insertion	0.999^**^	0.998^**^
Area of direct tibial insertion	1.000^**^	1.000^**^
Area of indirect tibial insertion	0.999^**^	1.000^**^
Area of total tibial insertion	0.999^**^	1.000^**^
Length of ACL	0.996^**^	0.981^**^

** Represents p < 0.001.

The means, standard deviations and ranges of the measured morphological parameters of the ACL are given in Table 2. Individual variation was recorded for all parameters, but the area of total femoral insertion and length of the ACL showed relatively low variation (both had a Std. Deviation < 15% × Mean value) in comparison to the other parameters. The mean value of the total area of femoral insertion site was 3.4 times greater than the CSA of the isthmus, and the area of the tibial side was 4.5 times greater than the isthmus CSA. The mean area of the direct femoral insertion site was smaller than that of the indirect insertion. However, the direct tibial insertion site had a larger area than the indirect site.

**Table 2:** Measurements of cross-sectional area (CSA) of ACL isthmus, area of ACL femoral insertion sites (direct, indirect, and total), area of ACL tibial insertion sites (direct, indirect, and total) and ACL lengths displayed as mean, standard deviation and range (min–max).

	**Mean**	**Std. deviation**	**Range**
CSA at ACL isthmus (mm^2^)	67.44	10.39	44.66–87.22
Area of direct femoral insertion (mm^2^)	88.98	16.68	66.30–127.72
Area of indirect femoral insertion (mm^2^)	138.75	24.89	92.92–177.56
Area of total femoral insertion (mm^2^)	227.73	22.41	188.09–258.41
Area of direct tibial insertion (mm^2^)	186.90	42.94	124.51–304.66
Area of indirect tibial insertion (mm^2^)	114.33	37.96	65.07–214.58
Area of total tibial insertion (mm^2^)	301.22	48.09	233.58–412.04
Length of ACL (mm)	26.11	3.50	21.01–35.88

Non-linear regression analysis showed significant correlations between 12 pairs of ACL morphologic parameters (Table 3), within which strong correlations (labeled as “^**^” in Table 3) were found between (i) CSA at ACL isthmus and total area of tibial insertion site; (ii) area of indirect femoral insertion site and the total area of femoral insertion site; (iii) area of direct tibial insertion site and the total area of tibial insertion site. Significant but not strong correlations (labeled as “^*^” in Table 3) were found between (i) area of direct femoral insertion site and area of indirect femoral insertion site; (ii) CSA at ACL isthmus and area of direct tibial insertion site; (iii) area of indirect tibial insertion site and area of total tibial insertion site; (iv) length of ACL and area of indirect tibial insertion site.

**Table 3:** Results of nonlinear regression analysis showing statistical significance (Sig.) and coefficient of determination (*R*^2^) representing consistency between the best-fit line and the raw data, with each measured parameter used as a single variation.

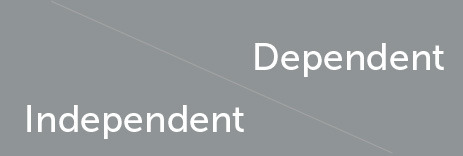		**CSA**	**Fdirect**	**Findirect**	**Ftotal**	**Tdirect**	**Tindirect**	**Ttotal**	**Length**
CSA	*R* ^2^		0.204	0.012	0.275	**0.427** ^*^	0.126	**0.374** ^**^	0.266
	Sig.		0.161	0.657	0.077	**0.012** ^*^	0.135	**0.005** ^**^	0.084
Fdirect	*R* ^2^	0.208		**0.251** ^*^	0.158	0.027	0.038	0.007	0.024
	Sig.	0.154		**0.029** ^*^	0.252	0.501	0.733	0.741	0.525
Findirect	*R* ^2^	0.076	**0.277** ^*^		**0.572** ^**^	0.099	0.027	0.130	0.062
	Sig.	0.534	**0.021** ^*^		**0.000** ^**^	0.189	0.498	0.328	0.304
Ftotal	*R* ^2^	0.091	0.070	**0.572** ^**^		0.068	0.018	0.035	0.119
	Sig.	0.210	0.274	**0.000** ^**^		0.280	0.585	0.443	0.361
Tdirect	*R* ^2^	0.194	0.136	0.102	0.067		0.121	**0.432** ^**^	0.134
	Sig.	0.059	0.311	0.183	0.285		0.144	**0.002** ^**^	0.124
Tindirect	*R* ^2^	0.119	0.004	0.091	0.172	0.107		**0.304** ^*^	**0.258** ^*^
	Sig.	0.149	0.790	0.467	0.222	0.172		**0.014** ^*^	**0.026** ^*^
Ttotal	*R* ^2^	**0.374** ^**^	0.045	0.042	0.201	**0.432** ^**^	**0.304** ^*^		0.012
	Sig.	**0.005** ^**^	0.690	0.402	0.165	**0.002** ^**^	**0.014** ^*^		0.906
Length	*R* ^2^	0.024	0.024	0.056	0.124	0.113	0.184	0.023	
	Sig.	0.528	0.525	0.328	0.347	0.160	0.067	0.831	

CSA, cross-sectional area of ACL isthmus; Fdirect, area of direct femoral insertion site; Findirect, area of indirect femoral insertion site; Ftotal, area of total femoral insertion site; Tdirect, area of direct tibial insertion site; Tindirect, area of indirect tibial insertion site; Ttotal, area of total tibial insertion site; Length, length of ACL.

* Represents p < 0.05.

** Represents p < 0.01.

Bold values present the outcomes with statistical significance.

In addition, significant correlations were found between compounded parameters of the ACL morphology (Table 4), including (i) CSA at ACL isthmus and the total area of direct insertion sites; (ii) CSA at ACL isthmus and the total area of all insertion sites; (iii) ratio between the total area of the femoral insertion site to CSA at ACL isthmus and ratio between the total area of the tibial insertion site to CSA at ACL isthmus.

**Table 4:** Results of nonlinear regression analysis showing statistical significance (Sig.) and coefficient of determination (*R*^2^) between compound parameters of ACL morphology.

**Independent**	**Dependent**	***R* ^2^**	***P*-value**
CSA	Ftotal + Ttotal	0.505^**^	0.001^**^
CSA	Fdirect + Tdirect	0.222^*^	0.042^*^
Ttotal/CSA	Ftotal/CSA	0.323^*^	0.044^*^

CSA, cross-sectional area of ACL isthmus; Fdirect, area of direct femoral insertion site; Ftotal, area of total femoral insertion site; Tdirect, area of direct tibial insertion site; Ttotal, area of total tibial insertion site.

* Represents p < 0.05.

** Represents p < 0.01.

**Figure 5:**
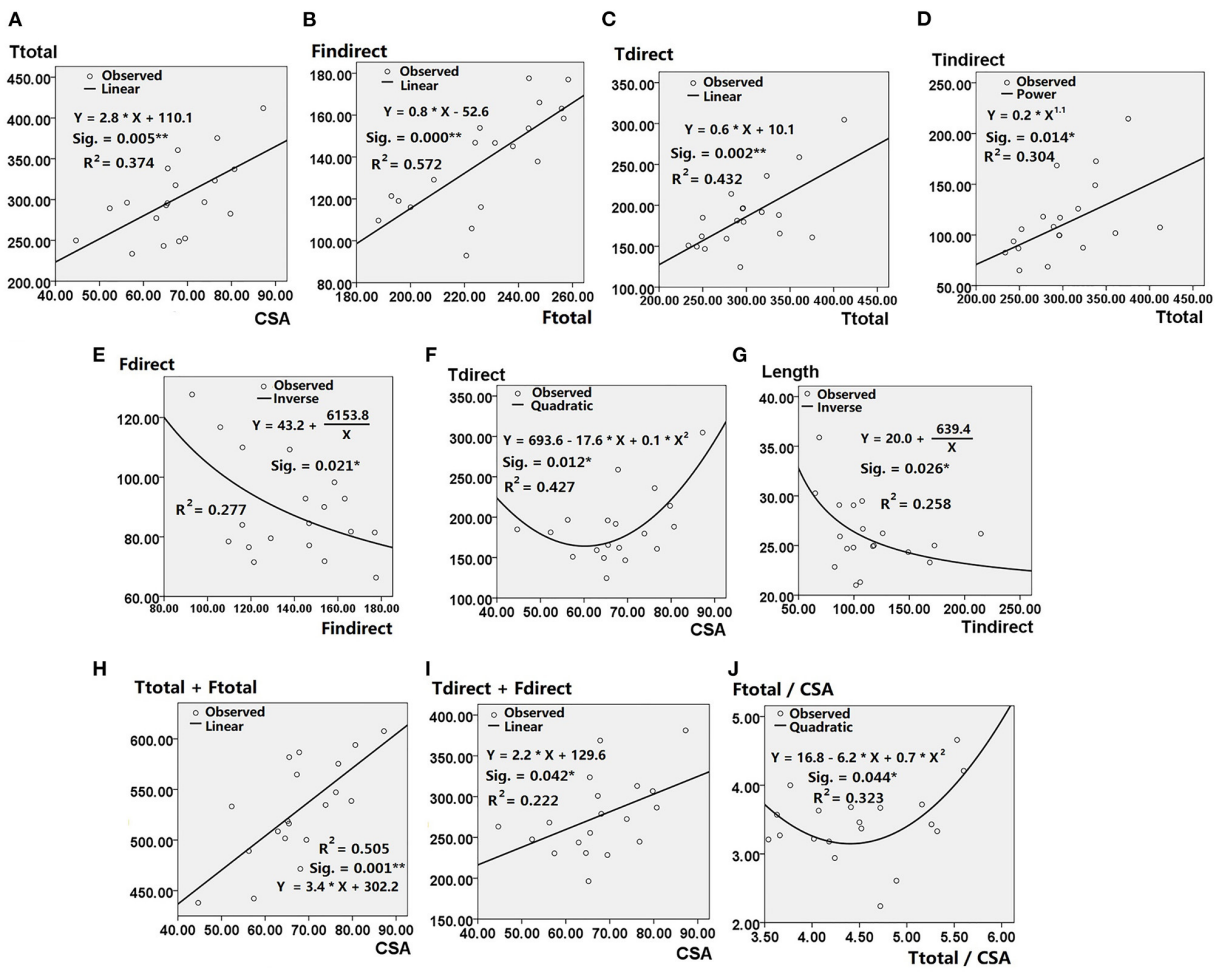
Results of nonlinear regression analysis showing statistical significance (Sig.), coefficient of determination (*R*^2^), Best-fit curves and the equations depicting correlation between **(A)** Ttotal and CSA; **(B)** Findirect and Ftotal; **(C)** Tdirect and Ttotal; **(D)** Tindirect and Ttotal; **(E)** Fdirect and Findirect; **(F)** Tdirect and CSA; **(G)** Length and Tindirect; **(H)** Ttotal + Ftotal and CSA; **(I)** Tdirect + Fdirect and CSA; **(J)** Ftotal/CSA and Ttotal/CSA. CSA, cross-sectional area of ACL isthmus; Fdirect, area of direct femoral insertion site; Findirect, area of indirect femoral insertion site; Ftotal, area of total femoral insertion site; Tdirect, area of direct tibial insertion site; Tindirect, area of indirect tibial insertion site; Ttotal, area of total tibial insertion site; Length, length of ACL. * Represents *p* < 0.05 when ** represents *p* < 0.01.

Graphs showing the non-linear curve for raw data, the best-fit curve and the equation representing the best-fit curve are shown in Figure 5. These graphs demonstrate the CSA at the ACL isthmus can predict the following morphological features of the ACL: (i) total area of tibial insertion site (Figure 5A); (ii) area of direct tibial insertion site (Figure 5F); (iii) total area of bony insertion sites (Figure 5H); (iv) total area of direct insertion sites (Figure 5I). Linear correlations with positive coefficients were found between the CSA at the isthmus and the aforementioned parameters, except for with the area of the direct tibial insertion site. The area of the direct tibial insertion site initially decreased with the CSA at the ACL isthmus but then increased once the CSA exceeded a certain value (around 60 mm^2^). Among all these best-fit linear equations, the coefficient was highest for the correlation between the CSA at the ACL isthmus and the total area of bone insertion sites.

Positive linear correlations were also shown between the following parameters: (i) total area of femoral insertion site and area of its indirect part (Figure 5B); (ii) total area of tibial insertion site and that of its direct part (Figure 5C); (iii) total area of tibial insertion site and that of its indirect part (Figure 5D).

Inverse correlations were shown between the following parameters: (i) area of direct and indirect femoral insertion site; (ii) area of indirect tibial insertion site and length of ACL (Figure 5G). The ratio of area of femoral insertion site to CSA at the ACL isthmus decreased with the ratio of area of tibial insertion site to CSA at the ACL isthmus, but increased when the latter exceeded a certain value (around 4.5) (Figure 5J).

## 4. Discussion

This study found significant and strong correlations between morphological features of a porcine ACL. The CSA at the ACL isthmus could predict the approximate area of the ACL bone insertion sites and the area of its tibial insertion site. Meanwhile, the area of the tibial insertion site could predict the area of its direct insertion, and the area of the femoral insertion site was correlated with the area of its indirect insertion. However, the area of the indirect tibial insertion site was only weakly correlated with the length of ACL, and the length of ACL may not be able to predict or be predicted by any of the other parameters.

This study used a porcine knee because numerous studies have shown a strong resemblance to the morphology and structure of human knees and associated ligaments. Hashemi et al. (6) reported a range of 35.1–103.6 mm^2^ for the CSA of the ACL isthmus in people, which was similar to the range recorded for the porcine ACL in this study (44.66–87.22 mm^2^). Other studies reported lower average values for the CSA of the ACL in human knees than porcine; 38.7 mm^2^ in Siebold's study (12) and 39.9 mm^2^ in Fujimaki's study (8), compared with 67.4 mm^2^ for porcine ACL measured in this study. The area of the ACL bone insertion site also shows individual variation between people (5, 8, 9, 12), ranging from 113 to 156 mm^2^ on the femoral side and 111–176 mm^2^ on the tibial side, but these values are still considerably smaller than that of the porcine ACL (228 mm^2^ for the femoral side and 301 mm^2^ for the tibial side). For both porcine and human ACL, the area of the tibial insertion site is generally larger than that of the femoral side, and the averaged areas of the two insertion sites were both over 3 times larger than the CSA at the ACL isthmus. Mochizuki et al. (7) measured the area of total and direct insertion sites for the femoral side and showed that the total area was almost 3 times that of its direct part (142.2 vs. 50.8 mm^2^), which was similar to the porcine ACL (227.7 vs. 89.0 mm^2^). The length of the porcine ACL compared well with that of a human ACL measured by Hashmi et al. (6) (21.01–35.88 vs. 23.80–33.65 mm). Overall, the cross-sectional area of a human ACL isthmus and the areas of its bone insertion sites are smaller than in pigs, but there are also important similarities between the two: the areas of bone insertion sites are considerably larger than the isthmus cross-sectional area, the area of the tibial insertion site is larger than that on the femoral side, and the area of the indirect femoral insertion site is significantly larger than that of the direct insertion site. These similarities support the suggestion that the correlations found in the morphology of porcine ACL might also exist in a human ACL.

On average, the areas of bone insertion sites were much larger than the CSA at the ACL isthmus (3.4 times for the femoral side and 4.5 times for the tibial side). The hourglass shape of the ACL substance might be a result of adaptation to provide sufficient rotational restraint to the knee joint while avoiding impingement of the midsubstance with the top surface of the femoral notch. The CSA at the ACL isthmus was found to strongly predict the total area of its bone insertion sites, implying that a robust ACL midsubstance may need a corresponding strong bony root to anchor it and reduce potential stress concentrations at the soft-hard tissue interface. The area of the femoral insertion site showed strong correlation with its indirect part but no significant correlation with its direct part. This may be because the indirect part occupied most of the area of the total insertion and was more representative of the total size. In contrast, the direct insertion on the tibial side occupied most of the total area thus it had stronger significant correlation with the total area of the tibial insertion site than the indirect part. The CSA at the ACL isthmus can predict the area of the tibial insertion site but cannot predict that of the femoral side. This may indicate that the tibial side predominantly increases its insertion area as the ACL midsubstance increases in volume. As shown in Table 2, individual differences in the area of the femoral insertion site (Std. Deviation = 10% × Mean) were relatively small in spite of the large variation on the tibial side (Std. Deviation = 16% × Mean) and the CSA at the ACL isthmus (Std. Deviation = 15% × Mean). This might be further reflected by the best-fit equations shown in Figure 5E that the area of the direct femoral insertion decreased as the indirect area increased, which is probably because of the relatively constant area of the total femoral insertion. Similarly, the weak correlation shown between the ACL length and other parameters might be caused by a relatively low and constant value for the ACL length (Std. Deviation = 13% × Mean). Several weak but significant correlations were found among the parameters in this study and are difficult to explain; inverse correlation between the area of indirect tibial insertion and ACL length, quadratic correlation between the CSA at the isthmus and area of direct tibial insertion, and quadratic correlation between the ratios of two bony insertions to the CSA at the ACL isthmus. These correlations should be further explored in future studies.

There are some limitations to this study that should be noted. (i) best-fit equations depicting the quantitative correlations between morphological features of the ACL were based on a porcine model, and thus parameters for a human ACL should be verified before applying to clinical practice; (ii) the anterior horn of the lateral meniscus was found to insert into the ACL substance and pass through the interface between tibial insertion sites of the anteromedial and posterolateral bundles of the ACL (Figure 1), which may result in a larger measured area of tibial insertion site. (iii) the porcine samples used in this study were male in 1 year old, thus the outcomes may apply to human male in younger age. Future studies may consider exploring the effect of age and sex on the morphological features of ACL.

Significant correlations between morphological features of porcine ACL in this study provide a basis for exploring corresponding parameters in a human ACL. This may be expanded to consider choosing suitable graft sizes when combined with reference dimensions such as the cross-sectional area of the ACL isthmus, areas of bone insertion sites and length of the ACL, instead of only referring to the size of ACL isthmus. Giving due consideration to other parameters may improve rotational stability and mitigate long-term osteoarthritis after ACL reconstruction.

## 5. Conclusion

The CSA at the ACL isthmus can linearly predict the total area of its bone insertion sites, and thus may be used as a reference measurement when choosing a suitable graft diameter for ACL reconstruction. The area of the indirect femoral insertion is more representative of the total area than the direct part, while the direct insertion is more representative for the tibial side. The length of the ACL has little correlation with the area of the isthmus cross-section and bone insertion sites, and thus should be evaluated independently before surgery when needed for reference.

## Data availability statement

The original contributions presented in the study are included in the article/supplementary material, further inquiries can be directed to the corresponding author.

## Ethics statement

The animal study was reviewed and approved by the Institutional Animal Care and Use Committee in Shanghai Jiao Tong University (IACUC APPROVAL No. 202101260).

## Author contributions

HW and C-KC conceived and designed the study and interpreted the data. HW, ZZ, Y-MZ, and QS collaborated in data collection. ZZ and HW processed and analyzed the data. All authors participated in preparation, revision and approval of the version to be published.
